# Clinical study on a skin stretching technique with adjustable external fixators to treat skin defects

**DOI:** 10.1097/MD.0000000000022144

**Published:** 2020-09-11

**Authors:** Gaofei Wang, Xiuli Zhang, Zhufeng Zhang, Zhuang Wei

**Affiliations:** a Department of Hand Surgery; b Department of Chest Surgery, China-Japan Union Hospital, Ji Lin University, Changchun, China.

**Keywords:** adjustable external fixators, skin defects, stretching skin

## Abstract

The aim of this study was to determine the effectiveness of a skin stretching technique with adjustable external fixators in treating skin defects.

Eighteen patients treated with a skin-stretching technique with adjustable external fixators for skin defects from April 2017 to October 2019 were included. Visual Analogue Scale (VAS) scores were collected during therapy. The skin defects gradually became smaller until they were completely resolved according to the blood flow of the affected limb and wound skin (the color, temperature, elasticity, and capillary response). The defect sizes ranged from 4 cm × 2 cm to 20 cm × 6 cm.

The 18 adjustable external fixators were dismantled in 2 to 9 days (mean, 4.05 days) after the operation, and the defects were completely closed and the sutures were removed after 2 to 3 weeks. The average VAS score was 5.97. The follow-up period was 4 to 12 months (mean, 6.3 months); 17 patients healed well with linear small scar, and no infections or patients of necrosis were observed. Sensory recovery was assessed using the Medical Research Council scale, and all the sensation scores were S3+. Eight patients were healed after the first stage. Nine patients were closed totally while small sinus or skin defect were observed after sutures were removed; 3 patients were healed after the second debridement, and 6 patients finally healed after the dressings were changed. Patellar osteomyelitis recurred in 1 patient who was transferred to the Orthopedic Department for further treatment, and a flap graft procedure was performed.

The operation was simple and obviously reduced the course of the disease, the costs, and the damage to the donor site, and it is also significantly superior to skin graft or flap transplantation procedures in terms of the resulting skin sensation, color, texture, elasticity, and appearance.

## 1 Introduction

Currently, skin grafts and flap surgeries are still widely used to treat large wounds.^[1–3]^ An advantage of skin grafts is the survival rate of the graft; the thinner the graft is, the higher the survival rate. A disadvantage is that the wound conditions are demanding; pressure needs to be applied to the skin and wound, they cannot slide relative to each other, and skin grafts cannot repair complex wounds exposing bones, joints or tendons. Skin grafts also lead to poor postoperative sensation, texture, elasticity, and configuration.^[3,4]^ A flap carries blood vessels and fat, which can cover wounds exposing bones, joints, and tendons. Disadvantages of flap surgeries include the fact that skin grafts or flaps are required to repair skin defects in donor areas and require a highly skilled doctor; other limitations include the need for skin histology tests, poor sensation, a bloated appearance, and high costs.^[3]^ The skin-stretching technique (SST) is used to gradually pull two edges of the wound to the middle and achieve complete wound closure in a short time, and it is achieved by specific skin characteristics, including a creep response, extensibility, viscoelasticity, and stress relaxation.^[1,5,6]^ Our study describes the use of adjustable external fixators as a skin-stretching device to treat 18 patients with skin defects from April 2017 to October 2019. Frequent adjustments were made according to the color, temperature, elasticity, and capillary responses of the edges of the skin, and satisfactory clinical results were achieved.

## 2 Materials and methods

This study was approved by the Ethics Committee of the China-Japan Union Hospital of Ji Lin University. A total of 18 patients were included in the study, and all patients signed informed consent forms. The patient group comprised 15 men and 3 women with an average age of 45.28 years (range: 34–75 years). The time from injury varied from 0 to 120 days, and the average time was 34.61 days.

This SST was applied to the dorsal side of 1 hand, 3 forearms, 2 knees, the anterior side of 3 calves, 2 ankles, 2 dorsal feet, the plantar area of 1 foot, 3 heels, and the medial area of 1 foot. The causes of the wounds included tumor ablations in 2 patients, trauma in 3 patients, and in 2 patients, there was exposure and damage to bone and soft tissue loss. Wound infections, exposed bone, and soft tissue loss were observed in 8 patients, frostbite and an exposed calcaneus were observed in 1 patient, surgical debridement for osteofascial compartment syndrome was performed in 1 patient, and there was 1 patient of a bite wound (Table 1). The defect sizes ranged from 4 cm × 2 cm to 20 cm × 6 cm (mean size, 8.08 cm × 3.61 cm).

**Table 1:**
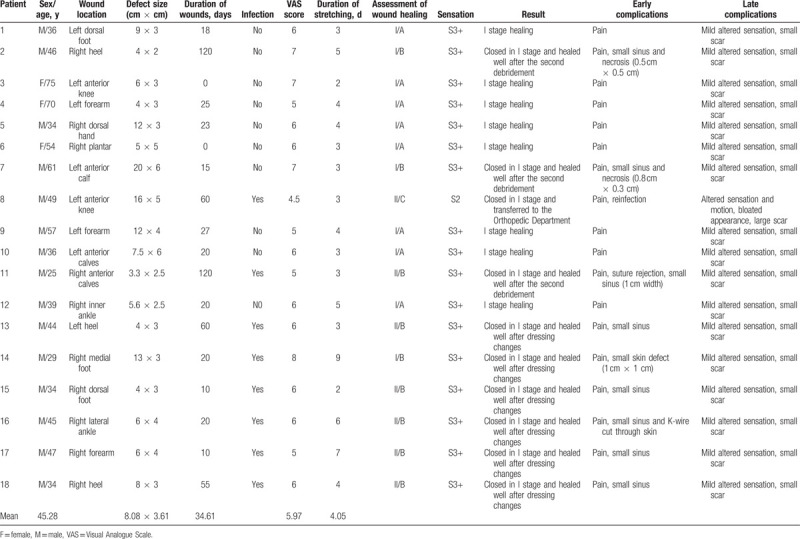
Patient summary.

### 2.1 Surgical technique

Bacterial cultures of all skin defects were assessed 3 consecutive times for negative results before the skin-stretching surgery. Surgery was performed under local infiltration anesthesia, epidural anesthesia, or general anesthesia. The wounds were cleansed thoroughly, the wound edges were excised partly, and blunt separation of the dermis was performed along the wound margin to 3 to 5 cm from the wound margin. Two K-wires that were 2.5 mm in diameter were inserted into the skin in a parallel manner 1 to 2 cm from each wound edge, passed through 2 to 3 cm of the dermis, removed from the skin and inserted back into skin 2 to 3 cm from the first insertion point. This operation was repeated until the entire length of the edges of the skin of the wound was fixed by K-wires, and 3 to 5 cm of the K-wires were remaining on the outside of the entry and exit points on the skin. Two adjustable external fixators (Zheng Tian Medical Inc, Tian Jin, China) or MiniRail Lengtheners (Orthofix Medical Inc, Italy), the size of which depended on the wounds, were fixed to the 2 sides of the K-wires at 1 to 2 cm from each entry and exit point. The screws of the external fixators were turned to reduce the wound edge gap in a parallel and even manner, whereas the blood flow of the affected limb and wound skin were monitored (the color, temperature, elasticity, and capillary response). The external fixators were tightened evenly and appropriately every day after the operation, and the skin defects gradually narrowed until they became completely closed. VAS scores were recorded during therapy. The adjustable external fixators were removed, and the wounds were sutured 2 to 9 days postoperatively (mean, 4.05 days). All patients underwent postoperative antibiotic treatment, and the sutures were removed after 2 to 3 weeks (Table 1).

## 3 Results

The pain during the stretching treatment was obvious in all patients. The average VAS score was 5.97. All 18 wounds closed completely, and the sutures were removed after 2 to 3 weeks. Postoperative follow-up ranged from 4 to 12 months (mean, 6.3 months). Seventeen patients healed acceptably without apparent complications, and no infections or patients of necrosis were observed. Sensory recovery was assessed using the Medical Research Council scale (MRC), and all the sensation scores were S3+. Eight patients were healed after the first stage, and all wounds healed satisfactorily without infections, a bloated appearance, or cutaneous necrosis. In the end, there was a 1 to 2 cm wide linear scar, and the state of healing was assessed to be I/A.^[7]^ Three patients were sutured completely while remained minor sinus and necrosis after sutures removing and then healed well after the second debridement; 1 patient had a preoperatively exposed calcaneus and infections, 1 patient had a long-term infection after tibial bone grafting, and the other had a tibia fracture before surgery. These 3 finally developed a 1 to 2 cm wide linear scar, and the state of healing was assessed to be I/B.^[7]^ In 6 patients, there were long-term infections before surgery; there was 1 patient of diabetes, but the wound culture results suggested there was no bacterial infection in the operation. Six patients of wounds were closed totally, whereas small sinus was reported in 5 patients and small skin defect (1 cm × 1 cm) in 1 patients after removed sutures and finally healed after dressing changes; only 1.5 to 2.5 cm wide linear scars were found, and the state of healing was assessed to be II/B.^[7]^ One patient was diagnosed with patellar osteomyelitis with primitive bone exposure, underwent this procedure after surgical debridement, and had negative results for the bacterial cultures of the skin defects. We observed a small amount of yellow sanies discharge from the sutured wound on postoperative day 3, and the bacterial cultures of the discharge showed staphylococcal infection. The state of healing of this patient was assessed to be II/C,^[7]^ and the patient was transferred to the Orthopedics Department for the removal of two-thirds of the infective patellar. Finally, a flap graft was performed. At the 10-month follow-up, the shape and active range of movement of the knee were obviously inferior to those of the contralateral knee, a large scar was observed and the level of sensation was recorded as S2 according to the MRC (Table 1).

## 4 Case report

**Figure 1:**
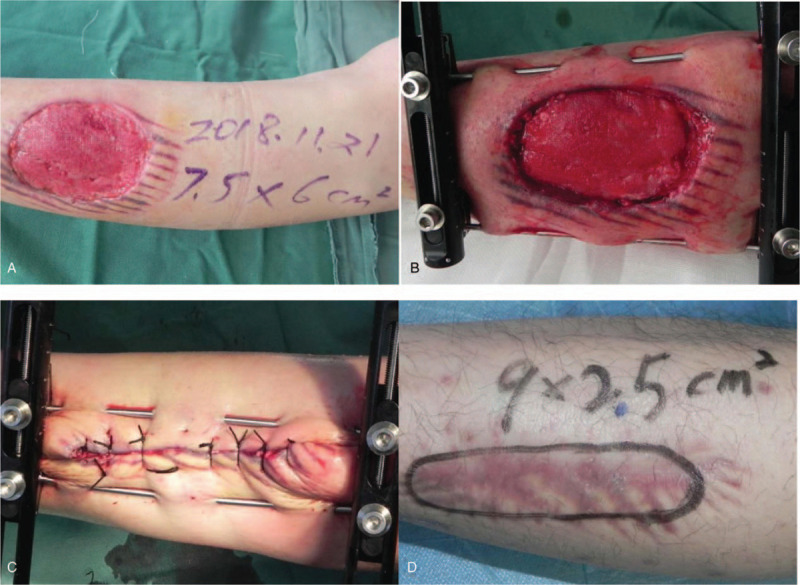
A 36-year-old male patient with a skin defect on the left leg for 20 days. (A) 7.5 cm × 6 cm skin defect. (B) Adjustable external fixators and K-wires stretching the skin. (C) Sutured skin and closed wound; the external fixators and K-wires were dismantled on the third postoperative day. (D) At the 4-month follow-up, the size of the scar was 9 cm × 2.5 cm, and the level of sensation was S3+.

A 36-year-old male patient with a left calf skin defect for 20 days underwent surgical debridement and negative-pressure VSD. There was a wound with a size of approximately 7.5 cm × 6 cm after VSD was performed. Adjustable external fixators and K-wires were used to stretch and close the skin, and the wounds were sutured. The external fixators and K-wires were dismantled on the third postoperative day. At the 4-month follow-up, the size of the scar was 9 cm × 2.5 cm, and the level of sensation was S3+ (Fig. 1).

## 5 Discussion

Barrer et al^[8]^ first used 3 bridging devices to stretch and close wounds in 1976. In 1987, Bashir^[9]^ fixed and encircled the wound with 4 K-wires, looped 2 silver wires around the cross points of the K wires, and then twisted silver wires to close the wounds. In 23 patients, the wounds gradually resolved and were sutured. There was necrosis on the edge of the skin where the K-wires crossed, a pause of 5 days was allowed before the twisting of the wires was resumed in 1 patient, and the final result was satisfactory. This method can provide a high stretching force, but a limitation is that it requires a long traction time (maximum 17 days) and easily damages the skin. Blomqvist and Steenfos^[10]^ described another method: several straps were placed subcutaneously and parallel under the skin defect through small channels on each side of the defect, 2 holding bars on each side of the skin defect were attached to the straps on both sides of the defect, and a locking device was attached. Thirty-two patients underwent this treatment. The wounds were sutured, and the devices were removed after 1 to 3 weeks. Pain was a complaint for 3 patients during the extension phase, and the device was removed before extension was completed in 2 patients. A skin-stretching device was reported by Hirshowitz et al^[5]^: 2 pins were threaded through the dermis on each side of the wound, 2 U-shaped arms under the surface of which were inward-facing sharp cutting hooks that engaged the pins through the skin surface overlying the pins, and a threaded screw was passed through the arms. The screw was turned, and the screw caused each side of the wound to be pulled closer together. Twenty-eight patients were treated by this method, which was employed over a duration of 20 minutes to 3 days and could be used for large skin defects (maximum size in this article was 27 cm × 15 cm). Wound infections were observed in 2 patients, and slight necrosis of the skin edges was observed in 1 patient. Qiqiang et al^[2]^ described 21 patients with skin and soft tissue defects in limbs that were repaired by a modified adjustable skin stretching and secure wound-closure system. The size of the skin defect area ranged from 4.0 cm × 2.5 cm to 21.0 cm × 10.0 cm. In 4 patients, the wounds closed directly after 1 stage; in 12 patients, the wounds were closed after continuous stretching for 5 to 14 days (mean, 10 days). The wounds were obviously reduced in 5 patients, and they were finally healed after the second skin graft procedure (4 patients) or flap repair procedure (1 patient). All 21 patients were followed up for 3 to 12 months (mean, 5.2 months). The wounds showed linear healing with small scar, and there were no patients of an invasive margin, poor blood flow, necrosis, or poor sensory function. The advantage of this method was that it can provide a strong force and be applied to large wounds. The pressure can also be flexibly regulated with this method, and the times of the adjustments are dependent on the color, temperature, capillary response, and swelling degree of the skin edge. The disadvantage is that it did not allow the skin and dermis to be free of wound margins to prevent blood problems, which reduced the speed of method and prolonged the duration for which the skin was pulled. Second, this method is not suitable for wounds in ankles, fingers, or the face.

All 18 patients’ adjustable external fixators and K-wires were dismantled, and the wounds were sutured 2 to 9 days postoperatively (mean, 4.05 days). All patients were followed-up for 4 to 12 months (mean, 6.3 months). Seventeen patients healed well without joint stiffness, pain, or deformity. There were no significant differences in the skin appearance, sensation, color, elasticity, or capillary filling test results compared to the opposite limb. The width of scar size ranged from 1 cm to 2.5 cm, and we believe it was caused by the stretching force, and in all patients, the level of sensation was S3+. Eight wounds with good condition (good shape, short exposure time, no infection, and diabetes) were stretched after negative results for 3 bacterial culture tests were obtained, or this method was applied directly after tumor excision, and the patients healed satisfactorily after the first stage. However, poor condition wounds due to long-term infection (subcutaneous or osteomyelitis) and exposure were observed in 9 patients, were sutured at the first stage and developed to small sinus or skin defect, finally healed well after secondary debridement or dressing changes. We supposed that although the results of 3 consecutive preoperative bacterial culture tests in longstanding and infective wounds were negative, the wounds still easily develop poor margin conditions, requiring a long wound healing period and develop large scars. There was sinus and necrosis in 3 patients after the wound was sutured, and those wounds were almost healed so that did not need stretching anymore. One patient (0.5 cm × 0.5 cm sinus and necrosis) was caused by frostbite with exposed calcaneus and infection before surgery and healed 2 weeks after the second debridement; 1 patient (1 cm wide sinus) was considered to be caused by suture rejection and a long-term infection after tibial bone grafting (>6 months) and healed completely after second suture; the other patient (0.8 cm × 0.3 cm sinus and necrosis) was considered to be caused by a tibia fracture and finally healed well after a secondary excision of dead bone. Small sinus appeared in 5 patients. Two patients were result from difficult shape, infection, and soft tissue loss, and 1 patient was caused by both a long-term heel wound infection (>4 months) and diabetes. In 1 patient, wound infection, necrosis, and poor skin conditions at the wound edges were caused by the K-wire cutting through the skin and affecting blood flow in the skin, as a wound in the lateral malleolus developed because of long-term tibial osteomyelitis (>20 years). In addition, the Achilles tendon was exposed (2 months) and there was an infection in 1 patient. Small skin defect (1 cm × 1 cm) was observed in 1 patient which was considered to be caused by fracture, difficult shape, and location (defects in the plantar arch and medial foot). Both joint and tendon were covered. All 6 patients postoperatively sutured wounds while remained small sinus or skin defect and finally showed satisfactory healing after dressing changes. One patient had been treated for patella fractures and patella osteomyelitis in the Orthopedic Department. After negative results for 3 consecutive bacterial culture tests were obtained, this patient underwent skin stretching. The adjustable external fixators were removed, and the wounds were sutured completely on postoperative day 3. Patellar osteomyelitis recurred on the 3^rd^ day, and the patient was transferred back to the Orthopedic Department for resection of two-thirds of the infective patella. For ultimate wound healing, a flap graft was used to fill the defect. At the 10-month follow-up, there was a significant difference between the 2 sides in the knee shape (bloated appearance and large scar) and range of motion. The level of sensation was S2. Two fracture patients’ external fixators were attached by belts to avoid the use of K-wires, and the external fixators pressed on the fracture to influence the fracture healing process. All patients felt pain throughout stretching skin (mean VAS was 5.97), we reduced the amplitude of each stretch while increased the times of adjustments and used some drugs such as Propacetamol or Ibuprofen to relieve their pain. Hirshowitz^[5]^ reported that 3 of 28 patients (10.7%) developed wound infections after the skin-stretching device was used and were finally cured by systemic antibiotics and local antibacterial treatments. Jiang et al^[11]^ reported that 110 patients had undergone a skin stretching operation. Pain was observed in 9 patients, infections were observed in 2 patients, and all of the wounds were closed after the anti-infective treatment and surgery were performed. Jin-Ming et al^[12]^ used an external tissue extender to treat 8 patients of soft tissue defects in the ankle and foot. One patient who underwent arthrodesis of the ankle had a skin defect with a size of 5 cm × 3.5 cm, and the treatment was considered to have failed at the final 6-month follow-up due to long-term glucocorticoid therapy, infections, and skin rupture.

This method is suitable for skin defects with or without exposed bones, joints, and tendons caused by trauma or incisions for osteofascial compartment syndrome or tumor resection. It is also suitable for skin defects of residual limbs. Before the operation, bacteria should be completely removed from the wound, and negative results for 3 consecutive bacterial culture tests should be obtained. It should be noted that a pair of external fixators can only stretch the wound in a straight direction intraoperatively, which is important for wound such as a fusiform wound with obvious long axes. For wounds with several axes that are not aligned, external fixators should be added along the additional axes. For wounds without major axes, such as acute triangular wounds, the shape should be altered by excisions or local rotation flaps. For wounds in different planes, the skin can be stretched from different directions multiple times, and the external fixation can be appropriately applied to stretch the skin edges to avoid compression of the skin. For large skin defects, this method can effectively reduce the wound surface and cover exposed bones, joints, tendons, and other important tissues, and then, skin grafts or skin flaps can be used to resolve the remaining skin defects. For several adjacent wounds, the skin bridge can be transferred by a rotating flap or be removed to transform multiple wounds into one wound. The distance between the K-wires and wound margins is suggested to be at least 1 to 2 cm to avoid cutting through skin. In 2009, Marquardt et al^[13]^ measured the expansion at the skin surface in vivo as well as the corresponding intracutaneous oxygen partial pressure and considered ischemia to be caused by irreversible vascular destruction, prolonged pressure that exceeds capillary perfusion pressure and ischemic tolerance of the tissue, or protracted angiospasm. The key point of skin stretching technique was to ensure the survival of affected limb and wound skin. The oxygen partial pressure is the most meaningful parameter for evaluating tissue ischemia, in addition to the oxygen saturation and blood flow. We believed that the stretching direction, load, and healing duration depend on different location (relaxation or elasticity), shape (regular or irregular, large or small), course (long-term or not), degree of infection (subcutaneous or osteomyelitis), age, sex, with or without systemic disease, and so on. We observed that the major complication of this method was pain during the stretching phase. Second, another complication was that the adjustable external fixators can support a heavy load but easily affect skin blood flow, cut through skin and cause necrosis. The contraindications were ischemic diseases of the limbs, poor skin conditions of the wound margin, and degloving skin defects of the hands and feet.

### 5.1 Limitations

There were several limitations in this study. First, the number of patients was small, and the lesions were not concentrated. Second, we determined that the standard of pulling skin did not affect the blood flow of the skin margin or limbs. However, there were no objective criteria, such as blood oxygen saturation, partial oxygen pressure, or load criteria.

## 6 Conclusion

In summary, this operation was simple and obviously reduced the course of the disease, costs, and damage to the donor site, and it was also significantly superior to skin graft procedures or flap transplantation in terms of the resulting skin sensation, color, texture, elasticity, and configuration.

## Acknowledgments

All authors thank to all patients involved in this study from the China-Japan Union Hospital of Ji Lin University.

## Author contributions

**Conceptualization:** Zhuang Wei, Gaofei Wang.

**Data curation:** Gaofei Wang.

**Formal analysis**: Gaofei Wang, Xiuli Zhang, Zhufeng Zhang.

**Investigation**: Gaofei Wang, Zhufeng Zhang.

**Methodology**: Zhuang Wei, Gaofei Wang.

**Project administration**: Zhuang Wei, Gaofei Wang.

**Resources**: Zhuang Wei, Xiuli Zhang.

**Software**: Gaofei Wang.

**Supervision**: Zhuang Wei, Gaofei Wang, Zhufeng Zhang.

**Validation**: Zhuang Wei, Gaofei Wang.

**Writing – original draft**: Gaofei Wang.

**Writing – review & editing**: Zhuang Wei, Gaofei Wang.
